# Safety of the concurrent treatment of dogs with Bravecto™ (fluralaner) and Scalibor™ protectorband (deltamethrin)

**DOI:** 10.1186/1756-3305-7-105

**Published:** 2014-03-19

**Authors:** Feli M Walther, Petr Fisara, Mark J Allan, Rainer K A Roepke, Martin C Nuernberger

**Affiliations:** 1 MSD Animal Health Innovation GmbH, Zur Propstei, 55270 Schwabenheim, Germany; 2 MSD Animal Health, 26 Artisan Road, Seven Hills, NSW 2172, Australia

**Keywords:** Bravecto^TM^, Fluralaner, Dog, Safety, Scalibor™, Deltamethrin

## Abstract

**Background:**

Bravecto™ (fluralaner; MSD Animal Health) is a novel systemic ectoparasiticide for dogs providing long-acting flea- and tick-control after a single oral dose. Scalibor™ Protectorband (deltamethrin; MSD Animal Health) is a collar often used to reduce sandfly feeding for leishmaniasis prevention. This study investigated the safety of the concurrent use of Bravecto^TM^ and Scalibor^TM^ Protectorband at the recommended dosage regimens.

**Findings:**

Throughout the study period of 24 weeks, there were no clinical findings related to the concurrent treatment with Bravecto™ in dogs fitted with Scalibor™ Protectorband at the recommended dosage regimen.

**Conclusions:**

Concurrent treatment with Bravecto™ in dogs fitted with Scalibor™ Protectorband is well tolerated.

## Findings

Bravecto™ (active ingredient: fluralaner) is a new systemically administered insecticidal and acaricidal product. Numerous studies including a recent field study in dogs have shown that a single fluralaner dose administered orally as chewable tablet provides flea and tick control for twelve weeks [1].

Scalibor™ Protectorband (active ingredient: deltamethrin) is a collar that provides an anti-feeding effect for up to 6 months against ectoparasites including phlebotomine sandflies and mosquitos [2]. Sandflies are the vectors of *Leishmania spp*.

To protect dogs from tick and flea infestations, as well as from sandfly bites, both products may be administered concurrently. This study was conducted in healthy dogs to confirm the safety of the concurrent use of Bravecto™ and Scalibor™ Protectorband at the recommended dosage regimens [2,3].

## Methods

The study was conducted in Queensland, Australia, after obtaining the authorization of the relevant regulatory authorities (Queensland Department of Agriculture, Fisheries and Forestry, approval no. CA 2013/06/701).

Twenty healthy male and female dogs of various breeds were randomly assigned to two study groups. On day 0, dogs of the treatment group were fitted a Scalibor™ Protectorband collar and received a Bravecto™ (fluralaner) chewable tablet while dogs of the control group remained un-treated. Dogs of the treatment group were administered Bravecto™ on a second occasion on day 84. The actual fluralaner doses administered were 27 - 50 mg/kg BW. As indicated on the product leaflet dogs were fed around the time of Bravecto™ treatment [3], since bioavailability of fluralaner is higher in fed dogs [4]. All dogs were carefully observed for general health during the first hour following treatment and were examined by a veterinarian at 6, 12, 24, 32, 48, 56, 72, 80 hours, and 4, 6, 8, 10 days after each Bravecto™ treatment. The veterinarian examined for abnormalities in behaviour, coat and skin including collar administration site, locomotion, respiration, eyes, ears, nose, oral cavity, mucous membranes, capillary refill time, pulse palpation, vomitus, feces and urine as present in pen, and any other visible abnormalities. The clinical observations were scheduled to cover the period of highest systemic fluralaner exposure [5] and the time Scalibor™ Protectorband delivers the full efficacy following application [2]. Therefore, clinical signs associated with the concurrent use would most likely be apparent at these time points. Veterinary examinations continued on study days 27, 55, 83, 111, 139 and 168 (examinations included assessment of abnormalities in behaviour, locomotion, auscultation of heart and lung, heart rate, respiratory rate, pulse palpation, mucous membranes, capillary refill time, abdominal palpation, superficial lymph nodes, skin including collar administration site, eyes, pupils, ears, nose, mouth, teeth, tongue, anus, vagina, penile orifice, mammary glands, testicles, joints, feet, pads, rectal temperature) and general health observations (observations of dogs in their pen including check of collar administration site) were performed on all dogs once to twice daily. The veterinary study investigator assessed all parameters recorded and all clinical findings for their relationship to Bravecto™ and/or Scalibor™ treatment. Body weights were recorded weekly.

At monthly intervals dogs in both groups received moxidectin orally at a minimum dose of 3 mcg/kg BW for heartworm prevention. No clinical findings were observed in treatment or control group dogs associated with moxidectin administration.

## Results and discussion

Throughout the 24-week study period, there were no clinical findings related to the concurrent treatment with Bravecto™ in dogs fitted with Scalibor™ Protectorband.

In the treatment group single incidences of minor and transient localized skin reactions were observed at the collar application site, which were considered to be due to the mechanical influence of the collar. These observations are not unexpected in dogs wearing collars and are consistent with the product leaflet [2]; none of the collars needed to be removed. Such findings have not been reported in studies where only Bravecto™ was administered [1,6]. No other clinical findings related to the use of either product alone, or to the concurrent use of Bravecto™ and Scalibor™ Protectorband were observed. There were no obvious changes in group mean bodyweights during the study (the mean body weight of treated group was 17.3 kg pre-treatment on day -1 and 18.1 kg on day 168).

## Conclusion

Concurrent treatment with Bravecto™ (fluralaner) in dogs fitted with Scalibor™ Protectorband (deltamethrin) is well tolerated.

## Competing interests

FMW, PF, MJA, RKAR and MCN are employees of Merck/MSD Animal Health.

## Authors’ contributions

FMW, PF, MJA, RKAR and MCN authored the study design, monitored the study and interpreted the results. All authors revised and approved the final version of the manuscript.
